# High-Resolution α-Glucosidase Inhibition Profiling Combined with HPLC-HRMS-SPE-NMR for Identification of Antidiabetic Compounds in *Eremanthus crotonoides* (Asteraceae)

**DOI:** 10.3390/molecules21060782

**Published:** 2016-06-16

**Authors:** Eder Lana e Silva, Jonathas Felipe Revoredo Lobo, Joachim Møllesøe Vinther, Ricardo Moreira Borges, Dan Staerk

**Affiliations:** 1 Instituto de Pesquisas de Produtos Naturais, Centro de Ciências da Saúde, Universidade Federal do Rio de Janeiro, Rio de Janeiro 21941-902, Brazil; eder_lls@hotmail.com (E.L.S.); jonnyuff@gmail.com (J.F.R.L.); ricardo_mborges@yahoo.com.br (R.M.B.); 2 Department of Drug Design and Pharmacology, Faculty of Health and Medical Sciences, University of Copenhagen, Universitetsparken 2, Copenhagen DK-2100, Denmark; jomq@elektro.dtu.dk

**Keywords:** diabetes, HPLC-HRMS-SPE-NMR, α-glucosidase, *Eremanthus crotonoides*

## Abstract

α-Glucosidase inhibitors decrease the cleavage- and absorption rate of monosaccharides from complex dietary carbohydrates, and represent therefore an important class of drugs for management of type 2 diabetes. In this study, a defatted ethyl acetate extract of *Eremanthus crotonoides* leaves with an inhibitory concentration (IC_50_) of 34.5 μg/mL towards α-glucosidase was investigated by high-resolution α-glucosidase inhibition profiling combined with HPLC-HRMS-SPE-NMR. This led to identification of six α-glucosidase inhibitors, namely quercetin (**16**), *trans*-tiliroside (**17**), luteolin (**19**), quercetin-3-methyl ether (**20**), 3,5-di-*O*-caffeoylquinic acid *n*-butyl ester (**26**) and 4,5-di-*O*-caffeoylquinic acid *n*-butyl ester (**29**). In addition, nineteen other metabolites were identified. The most active compounds were the two regioisomeric di-*O*-caffeoylquinic acid derivatives **26** and **29**, with IC_50_ values of 5.93 and 5.20 μM, respectively. This is the first report of the α-glucosidase inhibitory activity of compounds **20**, **26**, and **29**, and the findings support the important role of *Eremanthus* species as novel sources of new drugs and/or herbal remedies for treatment of type 2 diabetes.

## 1. Introduction

Type 2 diabetes (T2D) is a chronic metabolic disorder that constitutes a global health problem, being responsible for almost 90% of all cases of diabetes in adults [1]. In 2014, approximately 348 million people suffered from T2D worldwide, and this number is estimated to reach 439 million in 2030 [2,3]. Severe micro- and macrovascular complications in T2D are caused by postprandial hyperglycemia following α-glucosidase-catalyzed hydrolysis of dietary carbohydrates—but the hydrolysis and absorption of glucose can be reduced by α-glucosidase inhibitors [4,5]. Acarbose, voglibose and miglitol are currently accepted clinical drugs for treatment of TD2. However, several side effects have been reported [6,7], which makes the search for new α-glucosidase inhibitors of utmost importance. From this perspective, the very recent development of state-of-the-art high-resolution bioassays combined with hyphenation of high-performance liquid chromatography, high-resolution mass spectrometry, solid-phase extraction and nuclear magnetic resonance spectroscopy [8], *i.e.*, HR-bioassay/HPLC-HRMS-SPE-NMR has been an important tool for identification of α-glucosidase inhibitors from plant and food extracts [9,10,11,12,13,14]—including the development of dual and triple high-resolution inhibition profiling [15,16,17]. Furthermore, the HR-bioassay/HPLC-HRMS-SPE-NMR platform has successfully been applied for identification of α-amylase inhibitors [18], fungal plasma membrane H^+^-ATPase inhibitors [19], antioxidants [20] and monoamine oxidase A inhibitors [21].

In our ongoing search for antidiabetic compounds from natural sources, *Eremanthus crotonoides* (DC.) Sch. Bip. (Asteraceae) was included in our screening of extracts for α-glucosidase inhibitory activity. Asteraceae is one of the most important plant families concerning natural products-based treatment of diabetes [22]. The genus *Eremanthus* Less. comprises 27 species geographically restricted to the Brazillian Cerrado, but there are some exceptions, like *E. crotonoides* that can also be found in the Restinga [23]. Previous phytochemical analysis of the genus led to identification of flavonoids, quinic acid derivatives, terpenes and sesquiterpene lactones [23,24,25,26,27,28]. Despite the importance of Asteraceae as plant-based remedies for management of diabetes, there are no reports on investigations of the antidiabetic activity of *Eremanthus* species. Thus, in this study, we used high-resolution α-glucosidase inhibition profiling in combination with HPLC-HRMS-SPE-NMR for investigation of α-glucosidase inhibitors in *E*. *crotonoides*.

## 2. Results and Discussion

### 2.1. High-Resolution α-Glucosidase Inhibition Profiling of Extract

**Figure 1:**
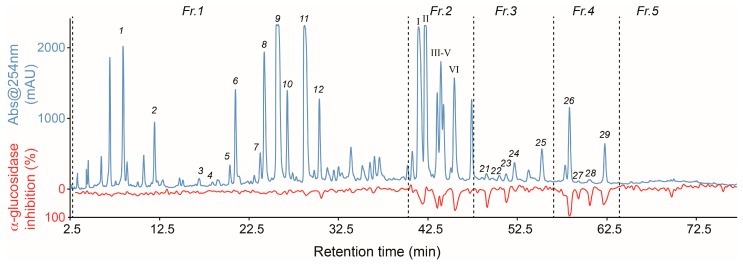
High-resolution α-glucosidase inhibition profile (red line) of defatted ethyl acetate extract of *E. crotonoides* shown underneath the HPLC chromatogram (blue line) at 254 nm. Fr.1–Fr.5 indicates the five regions collected by preparative-scale HPLC on C_18_ column for subsequent HPLC-HRMS-SPE-NMR analysis using analytical-scale pentafluorophenyl column

In our on-going screening programme for antidiabetic compounds from plants and foods, a defatted ethyl acetate extract of *E. crotonoides* showed an inhibitory concentration (IC_50_) of 34.5 μg/mL towards α-glucosidase, and was therefore selected for further investigation. Thus, 0.4 mg of the extract of *E*. *crotonoides* was subjected to analytical-scale reversed-phase HPLC, and the eluate in the retention time range from 3.0 to 78.0 min was fractionated into four 96-well microplates, yielding a resolution of 4.7 data points/min (the first column of each microplate was reserved for blanks and controls). This was followed by evaporation of the HPLC eluate from all wells, reconstitution in buffer containing 10% DMSO, and assessment of the α-glucosidase inhibitory activity of all wells (final concentration of DMSO during assay = 5%). The inhibitory activities (calculated as percentage inhibition) were plotted against the retention time from the microfractionation to give the high-resolution α-glucosidase inhibition profile (biochromatogram) shown in Figure 1. The HPLC chromatogram at 254 nm is shown with the blue line (top) and the high-resolution α-glucosidase inhibition profile shown with the red line at the bottom. The high resolution of the biochromatogram allows direct correlation of HPLC peaks and peaks in the biochromatogram—thereby pinpointing peaks corresponding to compounds with α-glucosidase inhibitory activity.

**Figure 2:**
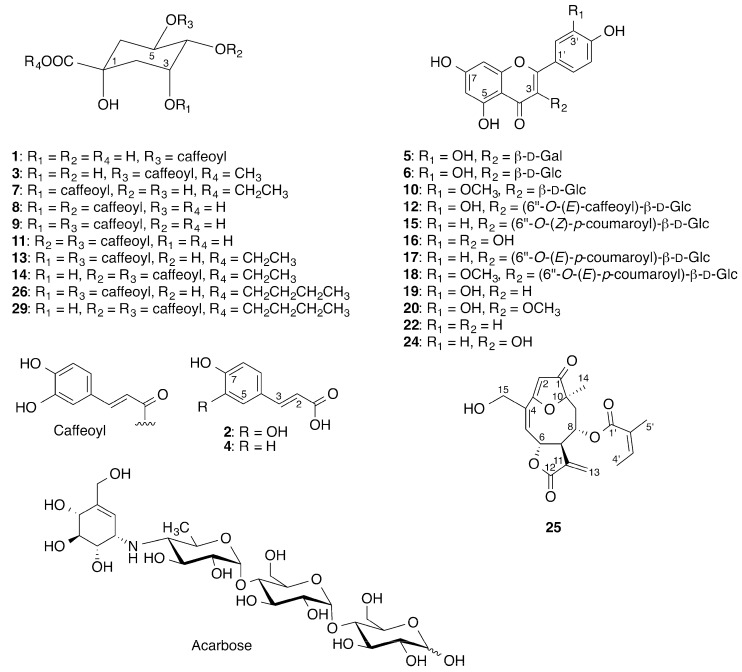
Acarbose and compounds identified in *Eremanthus crotonoides*.

The chromatogram shows several peaks in the range 5–65 min, with satisfactory separation of all major peaks, except in the range from 40 to 48 min. There seems to be minor α-glucosidase inhibitory activity correlated with peaks 11 and 12, but otherwise the majority of peaks correlated with strong α-glucosidase inhibitory activity are observed from 40 to 65 min. For dereplication purposes, the defatted extract was subjected to HPLC-HRMS-SPE-NMR analysis. This led to direct structural identification of 5-*O*-caffeoylquinic acid (**1**) [10], caffeic acid (**2**) [29], quercetin 3-*O*-β-d-glucoside (**6**) [10], 3-*O*-caffeoylquinic acid ethyl ester (**7**) [30], 3,4-di-*O*-caffeoylquinic acid (**8**) [31], 3,5-di-*O*-caffeoylquinic acid (**9**) [31], isorhamnetin-3-*O*-β-d-glucoside (**10**) [32], 4,5-di-*O*-caffeoylquinic acid (**11**) [10], quercetin-3-*O*-(6″-(*E*)-*O*-caffeoyl)-β-D-glucoside (**12**) [33], apigenin (**22**) [10], kaempferol (**24**) [34], centratherin (**25**) [35], 3,5-di-*O*-caffeoylquinic acid *n*-butyl ester (**26**) [36] and 4,5-di-*O*-caffeoylquinic acid *n*-butyl ester (**29**) [36] (Figure 2) directly from the extract, based on comparison of HRMS and NMR data obtained in the HPLC-HRMS-SPE-NMR mode (Table S1 in Supplementary Materials) with data from literature.

### 2.2. Identification of Inhibitory Constituents

The material eluted with the two major peaks I and II (Figure 1) in the retention time range 40–43 min were correlated with 44% α-glucosidase inhibition. The material eluted with the next three intense but overlapping peaks III–V displayed 52% to 62% α-glucosidase inhibition, whereas the material eluted with peak VI showed 69% α-glucosidase inhibition. Despite several attempts, it was not possible to develop an analytical-scale HPLC method for base-line separation of peaks I–V directly from the crude defatted extract. Thus, to investigate the material eluted with these peaks and some of the other minor peaks, the crude defatted extract of *E*. *crotonoides* was subject to preparative-scale reversed-phase HPLC to collect five major fractions (Fr.1–Fr.5 indicated in Figure 1).

Fraction Fr.1 was subjected to HPLC-HRMS-SPE-NMR analysis using an optimized separation method, which allowed identification of minor peaks 3–5 as 5-*O*-caffeoylquinic acid methyl ester (**3**) [29], *p*-coumaric acid (**4**) [37], quercetin 3-*O*-β-d-galactoside (**5**) [38] based on comparison of HRMS and NMR data obtained in the HPLC-HRMS-SPE-NMR mode (Table S1 in Supplementary Materials) with data from literature. Furthermore, a higher amount of **6**, **10** and **12** allowed acquisition of high-quality heteronuclear multiple bond correlation (HMBC) experiments which could not be directly obtained using the crude defatted extract.

**Figure 3:**
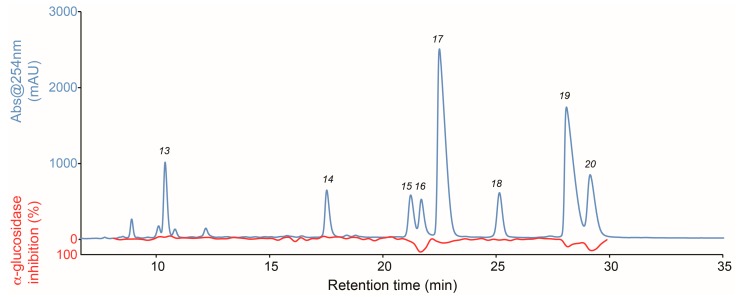
High-resolution α-glucosidase inhibition profile of fraction Fr.2 (red) shown underneath the HPLC chromatogram at 254 nm (blue).

Fraction Fr.2 was submitted to analytical-scale pentafluorophenyl (PFP) HPLC microfractionation, and an α-glucosidase biochomatogram was constructed for identification of α-glucosidase inhibitors. Figure 3 shows the chromatogram at 254 nm (blue line) and the high-resolution α-glucosidase inhibition profile obtained from microfractionation of Fr.2 (red line).

The biochromatogram shows that peaks 16, 17, 19, and 20 are correlated with α-glucosidase inhibitory activity of 82%, 23%, 32%, and 70%, respectively. For identification of the bioactive compounds, fraction Fr.2 was subjected to HPLC-HRMS-SPE-NMR analysis with trapping of peaks 13–20 after separation of ten successive injections; thereby ensuring high-quality NMR data for the trapped peaks. Based on HRMS as well as 1D and 2D NMR data (Table S1 in Supplementary Materials) obtained in the HPLC-HRMS-SPE-NMR mode, the peaks were identified as 3,5-di-*O*-caffeoylquinic acid ethyl ester (**13**) [39], 4,5-di-*O*-caffeoylquinic acid ethyl ester (**14**) [31], *cis*-tiliroside (**15**) [40], quercetin (**16**) [13], *trans*-tiliroside (**17**) [40], isorhamnetin-3-*O*-(6″-*O*-(*E*)-*p*-coumaroyl)-β-D-glucoside (**18**) [41], luteolin (**19**) [10] and quercetin 3-methyl ether (**20**) [34]. Peaks 15 and 18 were trapped in a separate HPLC-HRMS-SPE-NMR experiment with higher injection volumes in order to acquire HMBC data with sufficient high signal intensity (example of ^1^H and heteronuclear HSQC and HMBC for compound **15** are given in Supplementary Materials Figure S1a–c).

The identity of minor active peaks 21, 23, 27, and 28 could not be established from HPLC-HRMS-SPE-NMR analysis of the extract, *vide supra*, because the trapped fractions were impure and the analytes they contained were present in too low amounts. Despite several efforts, their identities could also not be established by HPLC-HRMS-SPE-NMR analysis after optimized separation of fractions Fr.3 and Fr.4.

### 2.3. Isolation and Pharmacological Evaluation of the α-Glucosidase Inhibitors

This is the first investigation of antidiabetic activity of an *Eremanthus* species, and it led to identification of six α-glucosidase inhibitors by high-resolution α-glucosidase inhibition profiling combined with HPLC-HRMS-SPE-NMR, *i.e.*, four flavonoids (**16**, **17**, **19**, and **20**) and two di-*O*-caffeoylquinic acid derivatives (**26** and **29**). Compounds **20**, **26** and **29** are known compounds, but their α-glucosidase inhibitory activity (70%, 92%, and 57%, respectively, as direct readouts from the biochromatogram) is reported for the first time. However, the percent inhibition in the biochromatogram are only relative inhibitions, since no information about the quantity of the individual constituents are available from these experiments. Thus, in order to isolate the α-glucosidase inhibitors for IC_50_ determination, fractions Fr.2 and Fr.4 were submitted to repeated analytical-scale HPLC fractionation using a PFP column. The flavonoids quercetin (**16**), *trans*-tiliroside (**17**), luteolin (**19**) and quercetin-3-methyl ether (**20**) were isolated from fraction Fr.2, whereas the di-*O*-caffeoylquinic acids derivatives 3,5-di-*O*-caffeoylquinic acid *n*-butyl ester (**26**) and 4,5-di-*O*-caffeoylquinic acid *n*-butyl ester (**29**) were isolated from fraction Fr.4.

**Figure 4:**
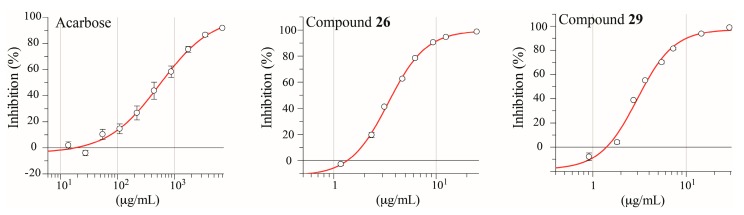
IC_50_ curves of Acarbose, **26** and **29**.

The isolated compounds and acarbose (reference compound) were tested for their inhibitory activity of yeast α-glucosidase, and IC_50_ curves for acarbose, **26** and **29** are shown in Figure 4 and IC_50_-values for all tested compounds are given in Table 1.

Compounds **16**, **17**, and **19** have all previously been reported as α-glucosidase inhibitors [42,43], but this is the first report of the α-glucosidase inhibitory activity of **20**, **26**, and **29**. Compounds **16**, **19**, **20**, **26**, and **29** all showed higher α-glucosidase inhibitory activity than the clinically approved antidiabetic drug acarbose (Table 1), which supports the use of *Eremanthus* as an antidiabetic herbal medicine.

Flavonoids are well-recognized α-glucosidase inhibitors, and in this work we report the α-glucosidase inhibitory activity of yet another flavonoid, quercetin-3-methyl ether (**20**). This compound has previously been reported as an inhibitor of human aldose reductase, another key therapeutic target in T2D [44]. Quercetin (**16**) showed higher α-glucosidase inhibitory activity than the new inhibitor **20** and luteolin (**19**), suggesting that methoxylation and hydroxylation at C-3 increase the inhibitory activity against yeast α-glucosidase. *trans*-Tiliroside (**17**) was less effective than flavonoids **16**, **19** and **20**. Previous reports have described that flavonoid glycosides show lower α-glucosidase inhibitiory activity than free aglycones [9,10].

The regioisomers 3,5-di-*O*-caffeoylquinic acid *n*-butyl ester (**26**) and 4,5-di-*O*-caffeoylquinic acid *n*-butyl ester (**29**) showed the highest inhibitory effects in the α-glucosidase assay. The use of plants that contain these metabolites for the treatment of T2D has been reported in folk medicine [36,45,46,47]. However, to the best of our knowledge, this is the first report regarding antidiabetic properties of compounds **26** and **29**. The similar non-esterified di-*O*-caffeoylquinic acids (compounds **8**, **9** and **11**) showed no or very low α-glucosidase inhibitiory activity as seen in the biochromatogram of *E*. *crotonoides* extract. Compound **11** is known as a weak inhibitor (IC_50_ > 100 μM) [31], which indicates that the *n*-butyl esterification in di-*O*-caffeoylquinic acid derivatives enhances the inhibitory activity against α-glucosidase.

## 3. Materials and Methods

### 3.1. Reagents

HPLC grade methanol and acetonitrile, petroleum ether, CDCl_3_, methanol-*d*_4_, dimethyl sulfoxide, acarbose, *p*-nitrophenol α-D-glucopyranoside (PNPG) and α-glucosidase type I (EC 3.2.20, from *Saccharomyces cerevisiae*, lyophilized powder) were purchased from Sigma-Aldrich (St. Louis, MO, USA). Methanol and ethyl acetate used for extraction were purchased from Tedia (Fairfield, OH, USA). Formic acid was obtained from Merck (Darmstadt, Germany) and water was prepared by a deionization and 0.22-µm membrane filtration system (Milipore, Billerica, MA, USA).

### 3.2. Plant Material and Sample Preparation

Leaves of *E. crotonoides* were collected in Restinga—Jurubatiba National Park, Rio de Janeiro, Brazil and identified by botanist Dr. Marcelo Guerra Santos. A voucher specimen has been deposited at the Herbarium of the Faculdade de Formaçãode Professores, Universidade Estadual do Rio de Janeiro, Brazil (M. Guerra Santos 2150). A portion (100 g) of the air-dried and powdered leaves was extracted with ethyl acetate (3 × 800 mL) using 24 min ultrasonication at room temperature, and the extract was subsequently dried under reduced pressure. The crude extract (4.0 g) was dissolved in H_2_O:MeOH (2:8) and defatted with petroleum ether. The defatted ethyl acetate extract was dissolved in methanol and filtered using Nylon Target Syringe Filters (0.45 μm pore size Thermo Scientific, Waltham, MA, USA) for further HPLC analysis.

### 3.3. High-Resolution α-Glucosidase Biochromatogram

Microfractionation was performed with an Agilent 1200 series instrument (Santa Clara, CA, USA) consisting of a G1316A quartenary pump, a G1322A degasser, a G1316A thermostatted column compartment, a G1315C photodiode-array detector, a G1364C fraction collector, and a G1367C high-performance auto sampler, controlled by Agilent ChemStation ver. B.03.02 software. The columns used were a Phenomenex C_18_(2) Luna (150 mm × 4.6 mm, 3 μm particle size, 100 Å pore size) and a Phenomenex PFP Kinetex (150 mm × 4.6 mm, 2.6 μm particle size, 100 Å pore size). For fractionation of the crude defatted ethyl acetate extract of *E. crotonoides* (injection: 4 μL; concentration: 100 mg/mL) the temperature was maintained at 40 °C and the flow rate at 0.5 mL/min. The solvents were a binary gradient mixture of water–acetonitrile (95:5 *v*/*v*) as eluent A and acetonitrile–water (95:5 *v*/*v*) as eluent B, both acidified with 0.1% formic acid. The following elution profile was used: 0 min, 10% B; 35 min, 26% B; 50 min, 35% B; 60 min, 40% B; 90 min, 100% B, and the eluate from 3 to 78 min was fractionated into four 96-well microplates, leading to a resolution of 4.7 data points per min. For fractionation of Fr.2 (injection: 2 μL; concentration: 33 mg/mL) the same conditions as described above were used, but with methanol and the PFP column instead of acetonitrile and the C_18_ column. The eluate from 8 to 30 min was fractionated into one 96-well microplate (resolution: 4.0 data points per min) using the following gradient: 0 min, 50% B; 3 min, 50% B; 34 min, 59% B; 35 min 100% B; 38 min, 100% B. The collected fractions were evaporated to dryness using a Savant SPD121P speed vacuum concentrator coupled with a RVT400 refrigerated vapor trap and an OFP-400 oil free pump (Holbrook, NY, USA). The α-glucosidase inhibitiory activity of the fractions was determined according to the previously described procedure [9]. Briefly, the content of each well was dissolved in 100 μL of 0.1 M phosphate buffer containing 10% of DMSO and added 80 μL of the α-glucosidase solution. After incubation at 28 °C for 10 min the reaction was initiated by addition of 20 μL of PNPG (10 mM in phosphate buffer) (final volume = 200 μL and final DMSO = 5%). The cleavage product of PNPG was monitored at 405 nm for 35 min using a Multiskan FC microplate photometer (Thermo Scientific, Waltham, MA, USA) and the percentage enzyme inhibition was calculated. The α-glucosidase inhibition of each well was plotted at their respective retention times underneath the HPLC chromatogram to obtain a high-resolution biochromatogram.

### 3.4. HPLC-HRMS-SPE-NMR Analysis

HPLC-HRMS-SPE-NMR analyses of crude defatted ethyl acetate extract and fractions of *E. crotonoides* were performed using an Agilent 1260 series chromatographic HPLC system consisting of a G1311B quaternary pump with built-in degasser, a G1329B autosampler, a G1316A thermostatted column compartment, and a G1315D photodiode-array detector. The crude defatted extract of *E. crotonoides* was separated using the same conditions (mobile phases, column, flow rate and elution gradient) as described above. Fraction Fr.2 was subjected to HPLC-HRMS-SPE-NMR analysis (injection: 2 μL; concentration of fraction Fr.2: 28 mg/mL) using the same conditions above, but with the following gradient 0 min, 50% B; 5 min, 50% B; 35 min, 63% B; 36 min, 100% B; 40 min, 100% B. Peaks 15 and 18 were trapped in a separate HPLC separation with a higher injection volume and concentration of fraction Fr.2 (injection: 7 μL; concentration of fraction Fr.2: 58 mg/mL) using the same conditions as described above for this fraction, but with the following gradient method: 0 min, 50% B; 30 min, 63% B; 31 min 100% B; 35 min, 100% B. Fraction Fr. 1 (injection: 8 μL; concentration of fraction Fr.1: 88 mg/mL) was analyzed with the same conditions as the extract of *E*. *crotonoides*, but with the following gradient method: 0 min, 10% B; 35 min, 24% B; 37 min, 100% B; 45 min, 100% B. For all separations, approximately 1% of the HPLC eluate was directed to a micrOTOF-Q II mass spectrometer (Bruker Daltonik GmbH, Bremen, Germany) equipped with an electrospray ionization (ESI) interface. Mass spectra were acquired in negative ion mode, using a drying temperature of 200 °C, capillary voltage of 4100 V, nebulizer pressure of 2.0 bar and a dry gas flow of 7 L/min. The other approximately 99% of the HPLC eluate was directed to the PDA detector and subsequently diluted with 1 mL/min of water by means of a Knauer Smartline Pump 100 (Knauer, Berlin, Germany), to a prospect 2 SPE-unit (Spark Holland, Emmen, The Netherlands). Before trapping, the cartridges were preconditioned with 500 μL of acetonitrile and subsequently equilibrated with 500 μL of water. Cumulative SPE trappings of the selected peaks on SPE cartridges (Hysphere GP phase, 10 × 2 mm i.d., from Spark Holland, Emmen, The Netherlands) were performed for 10 repeated separations for all HPLC analyses described above using absorption thresholds (254 and 320 nm) for trapping. Subsequently, the cartridges were dried with pressurized nitrogen gas for 45 min each and eluted into 1.7-mm o.d. NMR tubes (Bruker Biospin, Karlsruhe, Germany) with methanol-*d_4_* (final volume in tube 30 μL) using a Gilson 215 liquid handler (Gilson, Middleton, WI, USA) controlled by Prep Gilson software Version 1.2 (Bruker Biospin). MS measurements, HPLC separations and analyte trapping on SPE cartridges were controlled using Hystar version 1.2 software (Bruker Daltonik).

### 3.5. NMR Experiments

All NMR spectra were recorded in methanol-*d*_4_ at 300 K and ^1^H and ^13^C chemical shifts were referenced to the residual solvent signal (δ 3.31 and δ 49.00, respectively) (compound **25** was also analyzed in CDCl_3_). The experiments were performed with a Bruker Avance III system (^1^H operating frequency of 600.13 MHz) equipped with a Bruker SampleJet autosampler and a cryogenically cooled gradient inverse triple-resonance 1.7 mm TCI probe-head (Bruker Biospin) optimized for ^1^H and ^13^C observation. Bruker standard pulse sequences were used throughout this study. Icon NMR (version 4.2, Bruker Biospin) was used for controlling automated acquisition of NMR data (temperature equilibration to 300 K, optimization lock parameters, gradient shimming, and setting of receiver gain). NMR data processing was performed using topspin (version 3.1, Bruker Biospin). One-dimensional ^1^H-NMR spectra were acquired with 30°-pulses, 3.66 s inter-pulse intervals, 64 k data points and multiplied with an exponential function corresponding to line-broadening of 0.3 Hz prior to Fourier transform. Phase-sensitive DQF-COSY and NOESY spectra were recorded using a gradient-based pulse sequence with a 20 ppm spectral width and 2 k × 512 data points (processed with forward linear prediction to 1 k data points). Multiplicity-edited HSQC spectra were acquired with the following parameters: spectral width 20 ppm for ^1^H and 200 ppm for ^13^C, 2 k × 256 data points (processed with forward linear prediction to 1 k data points), and 1.0 s relaxation delay. HMBC spectra were optimized for ^n^*J*_C,H_ = 8 Hz and acquired using the following parameters: spectral width 20 ppm for ^1^H and 240 ppm for ^13^C, 2 k × 128 data points (processed with forward linear prediction to 1 k data points), and 1.0 s relaxation delay

### 3.6. Isolation and Evaluation of the Bioactive Compounds

The crude defatted ethyl acetate extract of *E. crotonoides* was subjected to reversed-phase preparative-scale HPLC fractionation (six injections of 900 μL; concentration: 100 mg/mL; flow: 20 mL/min) to collect five major fractions: Fr.1 (157 mg), Fr.2 (128 mg), Fr.3 (39 mg), Fr.4 (44 mg), and Fr.5 (78 mg). Preparative-scale fractionation was performed using an Agilent 1100 series instrument equipped with a multiple wavelength detector, an autosampler and two preparative-scale solvent delivery pumps. The column used was a Phenomenex C_18_(2) Luna (250 mm × 21.2 mm, 5 μm particle size, 100 Å pore size) and the gradient method was as follows: 0 min, 20% B; 27 min, 35% B; 37 min, 40% B; 40 min, 100% B; 46 min, 100% B; 47 min, 20% B; 52 min, 20% B using a binary mixture of water–acetonitrile (95:5 *v*/*v*) as eluent A and acetonitrile–water (95:5 *v*/*v*) as eluent B, both acidified with 0.1% formic acid. Fraction Fr.2 was subjected to PFP analytical-scale fractionation (40 injections of 3 μL each; concentration of fraction Fr.2: 78 mg/mL; collection threshold: 1050 mAU at 254 nm; column temperature: 40 °C; flow: 0.5 mL/min) using the same system of solvents as described above (but with methanol instead of acetonitrile) and the following gradient: 0 min, 50% B; 5 min, 50% B; 30 min, 58% B; 31 min, 100% B; 35 min, 100% B; 36 min, 50% B; 40 min, 50% B to yield compounds **16** (0.5 mg), **17** (1.3 mg), **19** (1.1 mg) and **20** (0.8 mg). Fraction Fr.4 was subjected to PFP HPLC analytical-scale purification (20 injections of 6 μL each; concentration of fraction Fr.4: 66 mg/mL; collection threshold: 450 mAU at 254 nm) using the same conditions as described for Fr.2 and the following gradient: 0 min, 55% B; 5 min, 55% B; 20 min, 75% B; 21 min, 100% B; 26 min, 100% B; 27 min, 55% B; 32 min, 55% B to yield compounds **26** (1.2 mg) and **29** (1.4 mg). The system used for fractionation of fractions Fr.2 and Fr.4 was the same as described in Section 3.3. The purity of the isolated compounds was assessed by ^1^H-NMR spectroscopy, and dilution series of the isolated compounds and acarbose (reference) were assessed for α-glucosidase inhibitory activity. The inhibition of the tested compounds was calculated using Equation (1).

%Inhibition = {(slope control − slope sample)/slope control)} × 100 (1)

These values were used for dose-response curves, and IC_50_ values were determined using GraFit (version 5.0.11) from Erithacus Software.

## 4. Conclusions

The present study demonstrated that *Eremanthus* species can be used as a natural source of α-glucosidase inhibitors. The HR-bioassay/HPLC-HRMS-SPE-NMR platform led to fast identification of three known compounds with already reported α-glucosidase inhibitory activity as well as three known compounds for which the α-glucosidase inhibitory activity is reported for the first time. Furthermore, use of the HPLC-HRMS-SPE-NMR platform led to identification of nineteen additional molecules. The results showed that the *n*-butyl group plays an important role for the strong α-glucosidase inhibitory activity of compounds **26** and **29** towards yeast α-glucosidase. These compounds are potential candidates for development of novel antidiabetic drugs and should be investigated in more detailed *in vitro* and *in vivo* studies.

## Figures and Tables

**Table 1:** Inhibitory activities of the bioactive compounds and reference compound acarbose.

Sample	IC_50_ (μM) ^a^
Acarbose ^b^	859.79 ± 0.09
**16**	7.19 ± 0.06
**17**	ND ^c,d^
**19**	59.64 ± 1.32
**20**	20.36 ± 1.30
**26**	5.93 ± 0.12
**29**	5.20 ± 0.30

^a^ Values represent means ± SD deviation of triplicate experiments; ^b^ Reference compound; ^c^ IC_50_ value not reached at concentration of 210 μM; ^d^ Inhibition at 210 μM = 4.5%.

## Supplementary Materials

Supplementary materials can be accessed at: http://www.mdpi.com/1420-3049/21/6/782/s1.
