# Supplementary material for: High-Resolution α-Glucosidase Inhibition Profiling Combined with HPLC-HRMS-SPE-NMR for Identification of Antidiabetic Compounds in Eremanthus crotonoides (Asteraceae)
Source: Molecules. 2016 Jun 16;21(6):782. doi: 10.3390/molecules21060782 (PMC6273868; doi:10.3390/molecules21060782)
Supplement: Supplementary file 1 [file molecules-21-00782-s001.pdf]

Supplementary Materials: High-Resolution α-Glucosidase Inhibition Profiling Combined with HPLC-HRMS-SPE-NMR for Identification of Antidiabetic Compounds in *Eremanthus crotonoides* (Asteraceae)

Eder Lana e Silva, Jonathas Felipe Revoredo Lobo, Joachim Møllesøe Vinther, Ricardo Moreira Borges and Dan Staerk

Table S1. ^{1}H NMR and MS data acquired in the HPLC-HRMS-SPE-NMR mode of 25 compounds from *E. crotonoides.*

Peak	Structure	^{1}H NMR δ (nH, m, *J* (in Hz))[a]	MS (*m*/*z*, molecular formula, ppm) [b]	Ref.
**1**	5-*O*-Caffeoylquinic acid	7.56 (1H, d, *J* = 16.0 Hz, H-7'), 7.05 (1H, d, *J* = 1.8 Hz, H-2'), 6.96 (1H, dd, *J* = 8.2, 1.8 Hz, H-6'), 6.78 (d, 1H, *J* = 8.2 Hz, H-5'), 6.26 (1H, d, *J* = 16.0 Hz, H-8'), 5.33 (1H, m, H-5), 4.16 (1H, m, H-3), 3.72 (1H, dd, *J* = 8.4, 3.0 Hz, H-4). H-2A/B and H-6A/B: 2.20 (2H, m) and 2.06 (2H, m)	353.0873 [M-H]$^-$ ($C_{16}H_{17}O_9^-$, Δ − 1.4 ppm)	[10]
**2**	Caffeic acid	7.52 (1H, d, *J* = 15.9 Hz, H-3), 7.03 (1H, d, *J* = 2.0 Hz, H-5), 6.92 (1H, dd, *J* = 8.2, 2.0, H-9), 6.77 (1H, d, *J* = 8.2 Hz, H-8), 6.21 (1H, d, *J* = 15.9 Hz, H-2)	179.0347 [M-H]$^-$ ($C_9H_7O_4^-$, Δ − 1.7 ppm)	[29]
**3**	5-*O*-Caffeoylquinic acid methyl ester	7.52 (1H, d, *J* = 16.2 Hz, H-7'), 7.04 (1H, d, *J* = 2.0 Hz, H-2'), 6.94 (1H, dd, *J* = 8.2, 2.0 Hz, H-6'), 6.78 (d, 1H, *J* = 8.2 Hz, H-5'), 6.22 (1H, d, *J* = 16.0 Hz, H-8'), 5.27 (1H, m, H-5), 4.13 (1H, m, H-3), 3.72 (1H, dd, *J* = 7.4, 3.2 Hz, H-4), 3.70 (3H, s, OCH_3). H-2A/B and H-6A/B: 2.21 (1H, dd, *J* = 13.6, 3.4 Hz), 2.15 (2H, m) and 2.00 (1H, m)	367.1017 [M-H]$^-$ ($C_{17}H_{19}O_9^-$, Δ − 4.9 ppm)	[29]
**4**	*p*-Coumaric acid	7.61 (1H, d, *J* = 15.8 Hz, H-3), 7.46 (2H, d, *J* = 8.4 Hz, H-6/H-8), 6.82 (2H, d, *J* = 8.4, H-9), 6.29 (1H, d, *J* = 15.8 Hz, H-2)	163.0396 [M-H]$^-$ ($C_9H_7O_3^-$, Δ − 3.0 ppm)	[37]
**5**	Quercetin-3-*O*-β-D-galactoside	7.83 (1H, d, *J* = 2.2 Hz, H-2'), 7.59 (1H, dd, *J* = 8.4, 2.2 Hz H-6'), 6.87 (1H, d, *J* = 8.4 Hz H-5'), 6.42 (1H, d, *J* = 2.0 Hz, H-8), 6.22 (1H, d, *J* = 2.0 Hz, H-6), 5.15 (1H, d, *J* =7.8 Hz, H-1''), 3.85 (1H, br d, *J* = 3.1, Hz, H-4''), 3.81 (1H, dd, *J* = 9.6, 7.8 Hz, H-2''), 3.64 (1H, dd, *J* = 11.0, 5.8 Hz, H-6''a), 3.54-3.57 (2H, m, H-3''/H-6''b), 3.47 (1H, td, *J* = 6.2, 0.8 Hz, H-5'')	463.0873 [M-H]$^-$ ($C_{21}H_{19}O_{12}^-$, Δ − 1.9 ppm)	[38]
**6**	Quercetin-3-*O*-β-D-glucoside	7.71 (1H, d, *J* = 2.0 Hz, H-2'), 7.58 (1H, dd, *J* = 8.4, 2.2 Hz H-6'), 6.87 (1H, d, *J* = 8.4 Hz H-5'), 6.41 (1H, d, *J* = 2.0 Hz, H-8), 6.22 (1H, d, *J* = 2.0 Hz, H-6), 5.24 (1H, d, *J* =7.7 Hz, H-1''), 3.70 (1H, dd, *J* = 12.0, 2.4 Hz, H-6''a), 3.57 (1H, dd, *J* = 12.0, 5.4 Hz, H-6''b), 3.48 (1H, dd, *J* = 9.0, 7.7 Hz, H-2''), 3.42 (1H, t, *J* = 9.0, H-3''), 3.35 (1H, t, *J* = 9.0, H-4''), 3.21 (1H, ddd, 9.6, 5.4, 2.4, H-5'')	463.0872 [M-H]$^-$ ($C_{21}H_{19}O_{12}^-$, Δ − 2.1 ppm)	[10]
**7**	3-*O*-Caffeoylquinic acid ethyl ester	7.52 (1H, d, *J* = 15.9 Hz, H-7'), 7.04 (1H, d, *J* = 2.0 Hz, H-2'), 6.94 (1H, dd *J* = 8.2, 2.0 Hz, H-6'), 6.79 (1H, d, *J* = 8.2 Hz, H-5'), 6.22 (1H, d, *J* = 15.9 Hz, H-8'), 5.27 (1H, m, H-5), 4.15 (3H, m, H-5/H-1''), 3.73 (1H, dd, *J* = 7.6, 3.2 Hz, H-4). H-2A/B and H-6A/B: 2.20 (1H, dd, *J* = 14, 3.6 Hz), 2.16 (2H, m), and 2.00 (1H, m). 1.24 (3H, t, *J* = 7.1 Hz, H-2'')	381.1181 [M-H]$^-$ ($C_{18}H_{21}O_9^-$, Δ − 2.6 ppm)	[30]
**8**	3,4-Di-*O*-caffeoylquinic acid	7.57/7.54 (2H, d, *J* = 16.0 Hz, H-7'/7''), 7.04/7.02 (2H, d, *J* = 2.0 Hz, H- 2'/2''), 6.93/6.87 (2H, dd, *J* = 8.0, 2.0 Hz, H-6'/6''), 6.77/6.73 (2H, d, *J* = 8.0 Hz, H-5'/5''), 6.28/6.25 (2H, d, *J* = 16.0 Hz, H-8'/8''), 5.63 (1H, m, H-3), 4.99 (1H, m, H-4), 4.37 (1H, m, H-5). H-2A/B and H-6A/B: 2.36 (1H, dd, *J* = 14.8, 2.8 Hz), 2.20 (1H, br d, *J* = 12.8) and 2.07-2.15 (2H, m)	515.1196 [M-H]$^-$ ($C_{25}H_{23}O_{12}^-$, Δ − 0.2 ppm)	[31]

**9**	3,5-Di-*O*-caffeoylquinic acid	7.62/7.57 (2H, d, *J* = 15.8 Hz, H-7'/7''), 7.07 (2H, br s, H- 2'/2''), 6.98/6.96 (2H, dd, *J* = 8.0, 2.0 Hz, H-6'/6''), 6.80/6.79 (2H, d, *J* = 8.0 Hz, H-5'/5''), 6.35/6.26 (2H, d, *J* = 15.8 Hz, H-8'/8''), 5.43 (1H, m, H-3), 5.38 (1H, m, H-5), 3.98 (1H, dd, *J* = 6.4, 3.1 Hz, H-4). H-2A/B and H-6A/B: 2.32 (1H, dd, *J* = 13.8, 4.0 Hz), 2.22-2.27 (2H, m) and 2.15 (1H, dd, *J* = 13.8, 7.0).	515.1200 $[M-H]^-$ ($C_{25}H_{23}O_{12}^-$, Δ + 1.0 ppm)	[31]
**10**	Isorhamnetin-3-*O*-β-D-glucoside	7.92 (1H, d, *J* = 2.0 Hz, H-2'), 7.60 (1H, dd, *J* = 8.4, 2.0 Hz H-6'), 6.91 (1H, d, *J* = 8.4 Hz H-5'), 6.42 (1H, d, *J*=2.0 Hz, H-8), 6.22 (1H, d, *J* = 2.0 Hz, H-6), 5.39 (1H, d, *J* = 7.3 Hz, H-1''), 3.95 (3H, s, OCH_3), 3.72 (1H, dd, *J* = 12.0, 2.2 Hz, H-6''a), 3.56 (1H, dd, *J* = 12.0, 5.6 Hz, H-6''b), 3.42-3.48 (2H, m, H-2''/H-3''), 3.35 (1H, m, H-4''), 3.24 (1H, ddd, *J* = 9.7, 5,6, 2.2 H-5'')	477.1034 $[M-H]^-$ ($C_{22}H_{21}O_{12}^-$, Δ − 0.8 ppm)	[32]
**11**	4,5-Di-*O*-caffeoylquinic acid	7.59/7.52 (2H, d, *J* = 15.8 Hz, H-7'/7''), 7.02/7.00 (2H, d, *J* = 2.0 Hz, H- 2'/2''), 6.92/6.90 (2H, dd, *J* = 8.2, 2.0 Hz, H-6'/6''), 6.75/6.74 (2H, d, *J* = 8.0 Hz, H-5'/5''), 6.28/6.18 (2H, d, *J* = 15.8 Hz, H-8'/8''), 5.62 (1H, m, H-5), 5.11 (1H, dd, *J* = 9.0, 3.0 Hz, H-4), 4.37 (1H, m, H-3). H-2A/B and H-6A/B: 2.21-2.31 (3H, m) and 2.11 (1H, dd, *J* = 14.2, 4.0)	515.1194 $[M-H]^-$ ($C_{25}H_{23}O_{12}^-$, Δ − 0.2 ppm)	[10]
**12**	Quercetin-3-*O*-(6''-*O*-*E*-caffeoyl)-β-D-glucoside	7.60 (1H, d, *J* = 2.2 Hz, H-2'), 7.56 (1H, dd, *J* = 8.4, 2.2 Hz, H-6'), 7.34 (1H, d, *J* = 15.8 Hz, H-3'''), 6.97 (1H, d, *J* = 2.2 Hz, H-5'''), 6.81 (1H, d, *J* = 8.4 Hz, H-5'), 6.80 (2H, m, H-9'' and H-8''') 6.32 (1H, d, *J* = 2.2 Hz, H-8), 6.15 (1H, d, *J* = 2.2 Hz, H-6), 6.04 (1H, d, *J* = 15.9, H-2'''), 5.23 (1H, d, *J* =7.7 Hz, H-1''), 4.28 (1H, dd, *J* = 12.0, 2.2 Hz, H-6''a), 4.19 (1H, dd, *J* = 12.0, 7.0 Hz, H-6''b), 3.51 (1H, t, *J* = 8.6 Hz, H-2''), 3.42-3.47 (2H, m, H-3''/H-5''), 3.35 (1H, m, H-4'')	625.1201 $[M-H]^-$ ($C_{30}H_{25}O_{15}^-$, Δ + 0.3 ppm)	[33]
**13**	3,5-Di-*O*-caffeoylquinic acid ethyl ester	7.62/7.55 (2H, d, *J* = 16.0 Hz, H-7'/7''), 7.06 (2H, d, *J* = 2.0 Hz, H- 2'/2''), 6.97/6.96 (2H, dd, *J* = 8.0, 2.0 Hz, H-6'/6''), 6.79 (2H, d, *J* = 8.0 Hz, H-5'/5''), 6.34/6.22 (2H, d, *J* = 16.0 Hz, H-8'/8''), 5.40 (1H, m, H-3), 5.30 (1H, m, H-5), 4.14 (2H, m, H-1''') 3.97 (1H, dd, *J* = 6.4, 3.0 Hz, H-4). H-2A/B and H-6A/B: 2.34 (1H, dd, *J* = 13.2, 4.0 Hz) and 2.13-2.31 (3H, m). 1.24 (3H, t, *J* = 7.2 Hz, H-2''')	543.1504 ($C_{27}H_{27}O_{12}^-$, Δ − 0.7 ppm)	[39]
**14**	4,5-Di-*O*-caffeoylquinic acid ethyl ester	7.60/7.51 (2H, d, *J* = 15.8 Hz, H-7'/7''), 7.03/7.00 (2H, d, *J* = 1.8 Hz, H- 2'/2''), 6.93/6.91 (2H, dd, *J* = 8.4, 1.8 Hz, H-6'/6''), 6.76 (2H, d, *J* = 8.0 Hz, H-5'/5''), 6.29/6.17 (2H, d, *J* = 15.8 Hz, H-8'/8''), 5.55 (1H, m, H-3), 5.11 (1H, dd, *J* = 8.0, 3.0 Hz, H-4), 4.34 (1H, m, H-5), 4.15 (2H, m, H-1'''). H-2A/B and H-6A/B: 2.33 (1H, dd, *J* = 13.8, 3.2 Hz), 2.21-2.30 (2H, m) and 2.09 (1H, dd, *J* = 13.8, 6.2 Hz). 1.26 (3H, t, *J* = 7.2 Hz, H-2''')	543.1495 ($C_{27}H_{27}O_{12}^-$, Δ − 2.4 ppm)	[31]
**15**	*cis*-Tiliroside	7.96 (2H, d, *J* = 8.8 Hz, H-2'/H-6'), 7.50 (2H, d, *J* = 8.6 Hz, H-2'''/H-6'''), 6.83 (2H, d, *J* = 8.8 Hz, H-3'/ H-5'), 6.70 (1H, d, *J* = 12.6 Hz, H-7'''), 6.68 (2H, d, *J* = 8.6 Hz, H-3'''/H-5'''), 6.33 (1H, d, *J* = 2.0 Hz, H-8), 6.20 (1H, d, *J* = 2.0 Hz, H-6), 5.51 (1H, d, *J* = 12.6 Hz, H-8'''), 5.20 (1H, d, *J* =7.3 Hz, H-1''), 4.21 (1H, dd, *J* = 11.6, 2.6 Hz, H-6''a), 4.17 (1H, dd, *J* = 11.6, 6.4 Hz, H-6''b), 3.40-3.45 (3H, m, H-2''/ H-3''/ H-5''), 3.28 (1H, t, *J* = 9.2 Hz, H-4'')	593.1298 ($C_{30}H_{25}O_{13}^-$, Δ − 0.5 ppm)	[40]
**16**	Quercetin	7.73 (1H, d, *J* = 2.2 Hz, H-2'), 7.64 (1H, dd, *J* = 8.2, 2.2 Hz H-6'), 6.89 (1H, d, *J* = 8.2 Hz H-5'), 6.40 (1H, d, *J*= 2.0 Hz, H-8), 6.19 (1H, d, *J* = 2.0 Hz, H-6)	301.0353 ($C_{15}H_9O_7^-$, Δ − 0.3 ppm)	[13]
**17**	*trans*-Tiliroside	7.98 (2H, d, *J* = 8.8 Hz, H-2'/H-6'), 7.39 (1H, d, *J* = 15.6 Hz, H-7'''), 7.30 (2H, d, *J* = 8.4 Hz, H-2'''& H-6'''), 6.82 (2H, d, *J* = 8.8 Hz, H-3' & H-5'), 6.79 (2H, d, *J* = 8.4 Hz, H-3''' & H-5'''), 6.32 (1H, d, *J* = 2.0 Hz, H-8), 6.14 (1H, d, *J* = 2.0 Hz, H-6), 6.07 (1H, d, *J* = 15.6 Hz, H-8'''), 5.24 (1H, d, *J* =7.3 Hz, H-1''), 4.30 (1H, dd, *J* = 11.8, 2.2 Hz, H-6''a), 4.19 (1H, dd, *J* = 11.8, 6.6 Hz, H-6''b), 3.43-3.49 (3H, m, H-2''/H-3''/H-5''), 3.33 (1H, m, H-4'')	593.1303 ($C_{30}H_{25}O_{13}^-$, Δ + 0.3 ppm)	[40]

**18**	Isorhamnetin-3-*O*-(6''-*O*-*E*-*p*-coumaroyl)-β-D-glucoside	7.85 (1H, d, *J* = 2.0 Hz, H-2'), 7.55 (1H, dd, *J* = 8.5, 2.0 Hz, H-6'), 7.37 (1H, d, *J* = 16.0 Hz, H-7'''), 7.30 (2H, d, *J* = 8.5 Hz, H-2'''/H-6'''), 6.85 (1H, d, *J* = 8.5 Hz, H-5'), 6.80 (2H, d, *J* = 8.5 Hz, H-3'''/H-5'''),), 6.31 (1H, d, *J*= 2.0 Hz, H-8), 6.15 (1H, d, *J* = 2.0 Hz, H-6),), 6.05 (1H, d, *J*= 16.0 Hz, H-8'''), 5.32 (1H, d, *J* =7.5 Hz, H-1''), 4.28 (1H, dd, *J* = 11.9, 3.0 Hz, H-6''a), 4.25 (1H, dd, *J* = 11.9, 6.2 Hz, H-6''b), 3.91 (3H, s, OCH_3), 3.45-3.52 (3H, m, H-2''/H-3''/H-5''), 3.33 (1H, m, H-4'')	623.1395 ($C_{31}H_{27}O_{14}^-$, Δ − 1.8 ppm)	[41]
**19**	Luteolin	7.38 (2H, m, H-2'/H-6'), 6.90 (1H, d, *J* = 8.8 Hz H-5'), 6.54 (1H, s, H-3), 6.44 (1H, d, *J* = 2.2 Hz, H-8), 6.21 (1H, d, *J* = 2.2 Hz, H-6).	285.0405 ($C_{15}H_9O_6^-$, Δ + 0.01 ppm)	[10]
**20**	Quercetin 3-methyl ether	7.62 (1H, d, *J* = 2.4 Hz, H-2'), 7.53 (1H, dd, *J* = 8.4, 2.4 Hz H-6'), 6.91 (1H, d, *J* = 8.4 Hz, H-5'), 6.40 (1H, d, *J*= 2.0 Hz, H-8), 6.21 (1H, d, *J* = 2.0 Hz, H-6), 3.78 s (OCH_3)	315.0507 ($C_{16}H_{11}O_7^-$, Δ − 0.9 ppm)	[34]
**22**	Apigenin	7.85 (2H, d, *J* = 8.8 Hz, H-2'/H-6'), 6.94 (2H, d, *J* = 8.8 Hz, H-3'/H-5'), 6.60 (1H, s, H-3), 6.47 (1H, d, *J* = 2.0 Hz, H-8), 6.22 (1H, d, *J* = 2.0 Hz, H-6)	269.0450 ($C_{15}H_9O_5^-$, Δ − 1.8 ppm)	[10]
**24**	Kaempferol	8.08 (2H, d, *J* = 8.8 Hz, H-2'/H-6'), 6.91 (2H, d, *J* = 8.8 Hz, H-3'/H-5'), 6.40 (1H, d, *J* = 1.8 Hz, H-8), 6.19 (1H, d, *J* = 1.8 Hz, H-6)	285.0400 ($C_{15}H_9O_6^-$, Δ − 1.7 ppm)	[34]
**25**	Centratherin[c]	6.29 (1H, m, H-5), 6.23 (1H, d, *J* = 3.1 Hz, H-13B), 6.09 (1H, qq, *J* = 7.4, 1.5 Hz, H-3'), 5.81 (1H, s, H-2), 5.45 (1H, d, *J* = 2.7, H-13A), 5.37 (1H, m, H-6), 4.54 dt (1H, dt, *J* = 12, 2.2, H-8), 4.40 (2H, m, H-15), 3.77 (1H, m, H-7), 2.49 (1H, dd, *J* = 13.8, 12.0, H-9A), 2.32 (1H, dd, *J* = 13.8, 1.9, H-9B), 1.89 (3H, dq, *J* = 7.4, 1.4, H-4'), 1.78 (3H, pentet, *J* = 1.4, H-5'), 1.54 (3H, s, H-14)	373.1287 ($C_{15}H_9O_6^-$, Δ − 1.6 ppm)	[35]
**26**	3,5-Di-*O*-caffeoylquinic acid *n*-butyl ester	7.62/7.54 (2H, d, *J* = 16.0 Hz, H-7'/7''), 7.07/7.06 (2H, d, *J* = 2.0 Hz, H- 2'/2''), 6.97 (2H, dd, *J* = 8.0, 1.8 Hz, H-6'/6''), 6.81/6.79 (2H, d, *J* = 8.2 Hz, H-5'/5''), 6.34/6.22 (2H, d, *J* = 16.0 Hz, H-8'/8''), 5.41 (1H, m, H-5), 5.29 (1H, m, H-3), 4.08 (2H, m, H-1''), 3.98 (1H, m, H-4), 2.12-2.19 and 2.33-2.36 (4H, m, H-2/H-6), 1.60 (2H, m, H-2''), 1.33 (2H, m, H-3''), 0.87 (3H, t, *J* = 7.4 Hz, H-4'')	571.1812 ($C_{29}H_{31}O_{12}^-$, Δ − 1.5 ppm)	[36]
**29**	4,5-Di-*O*-caffeoylquinic acid *n*-butyl ester	7.60/7.51 (2H, d, *J* = 16.0 Hz, H-7'/7''), 7.03/7.01 (2H, d, *J* = 2.2 Hz, H- 2'/2''), 6.96/6.90 (2H, dd, *J* = 8.2, 2.0 Hz, H-6'/6''), 6.75 (2H, d, *J* = 8.2 Hz, H-5'/5''), 6.28/6.17 (2H, d, *J* = 15.8 Hz, H-8'/8''), 5.55 (1H, m, H-3), 5.10 (1H, dd, *J* = 8.0, 3.0 Hz, H-4), 4.35 (1H, m, H-5), 4.10 (2H, m, H-1''), 2.27 (3H, m, H-2A/2B and H-6A), 2.08 (1H, ddd, *J* = 14.0, 6.8, 0.8 Hz, H-6B), 1.65 (2H, m, H-2''), 1.36 (2H, sextet, *J* = 7.2 Hz, H-3''), 0.92 (3H, t, *J* = 7.2 Hz, H-4'')	571.1807 ($C_{29}H_{31}O_{12}^-$, Δ − 2.4 ppm)	[36]

[a] ^1H resonance frequency 600.13 MHz. Spectra acquired at 300 K in methanol-d_4. [b] MS spectra acquired in negative ion mode. [c] ^1H NMR in $CDCl_3$.

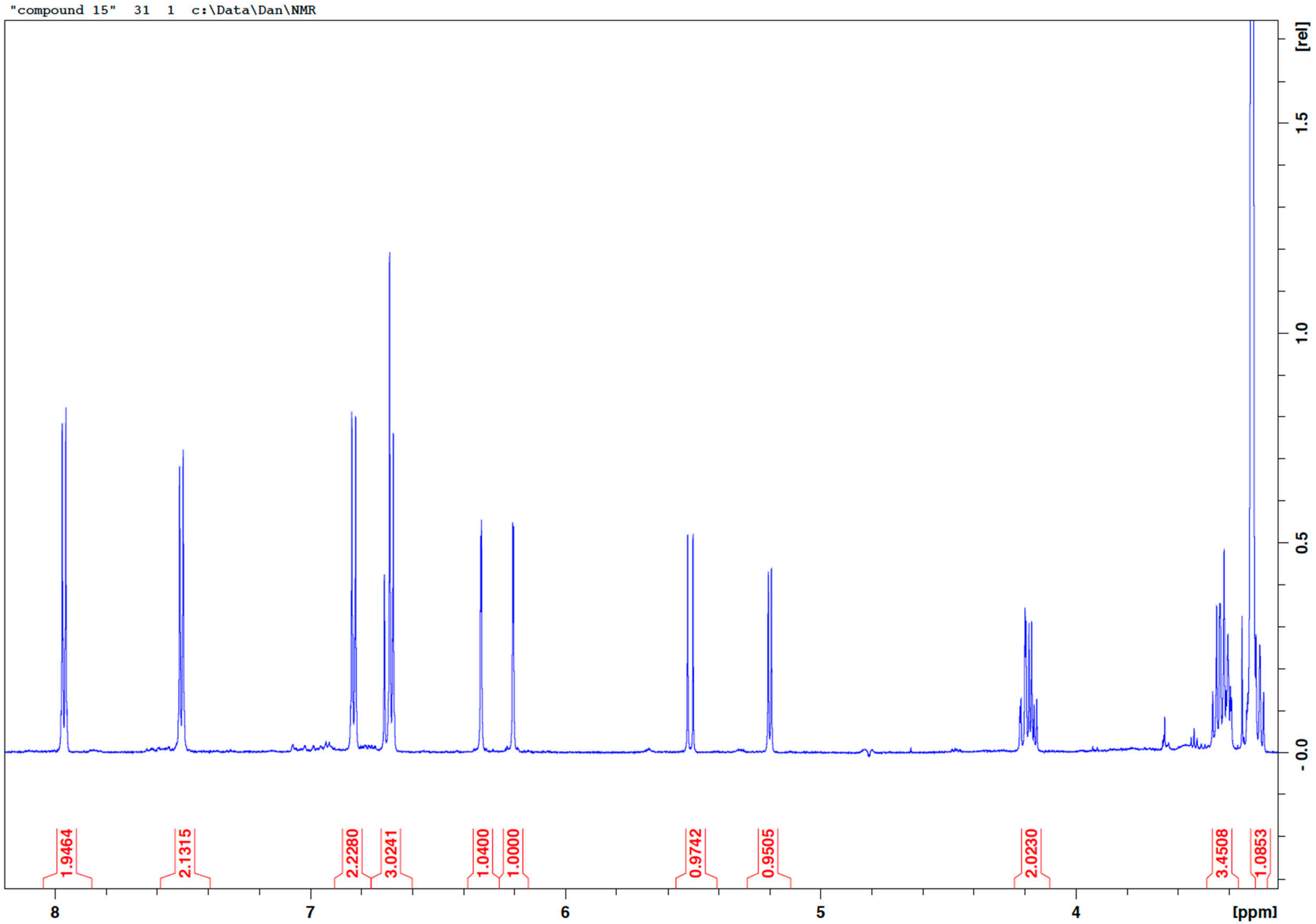
"compound 15" 31 1 c:\Data\Dan\NMR
[rel]
1.5
1.0
0.5
0.0
1.9464
2.1315
2.2280
3.0241
1.0400
1.0000
0.9742
0.9505
2.0230
3.4508
1.0853
8
7
6
5
4
[ppm]

(**a**)

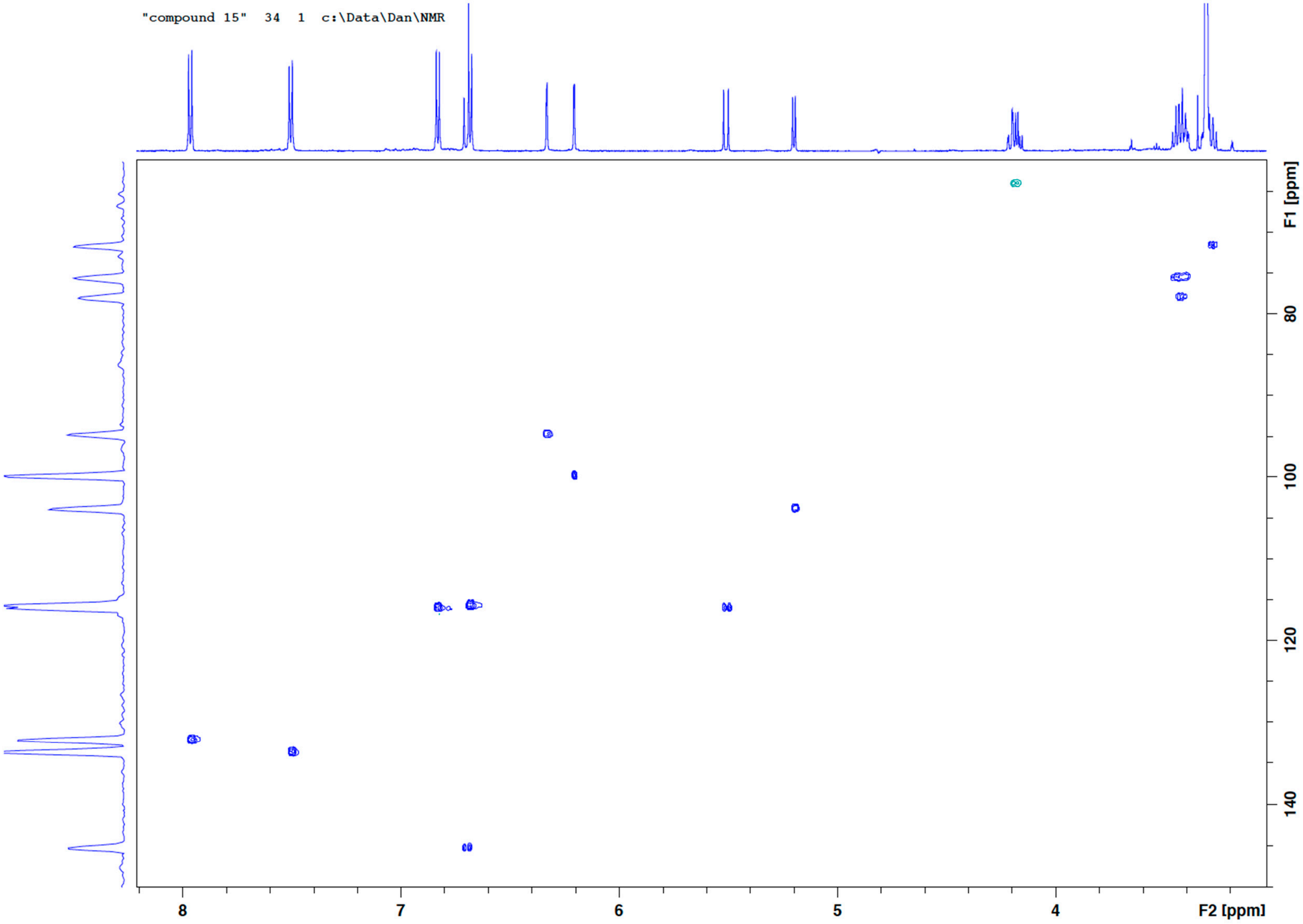
"compound 15" 34 1 c:\Data\Dan\NMR
F1 [ppm]
80
100
120
140
8
7
6
5
4
F2 [ppm]
(**b**)

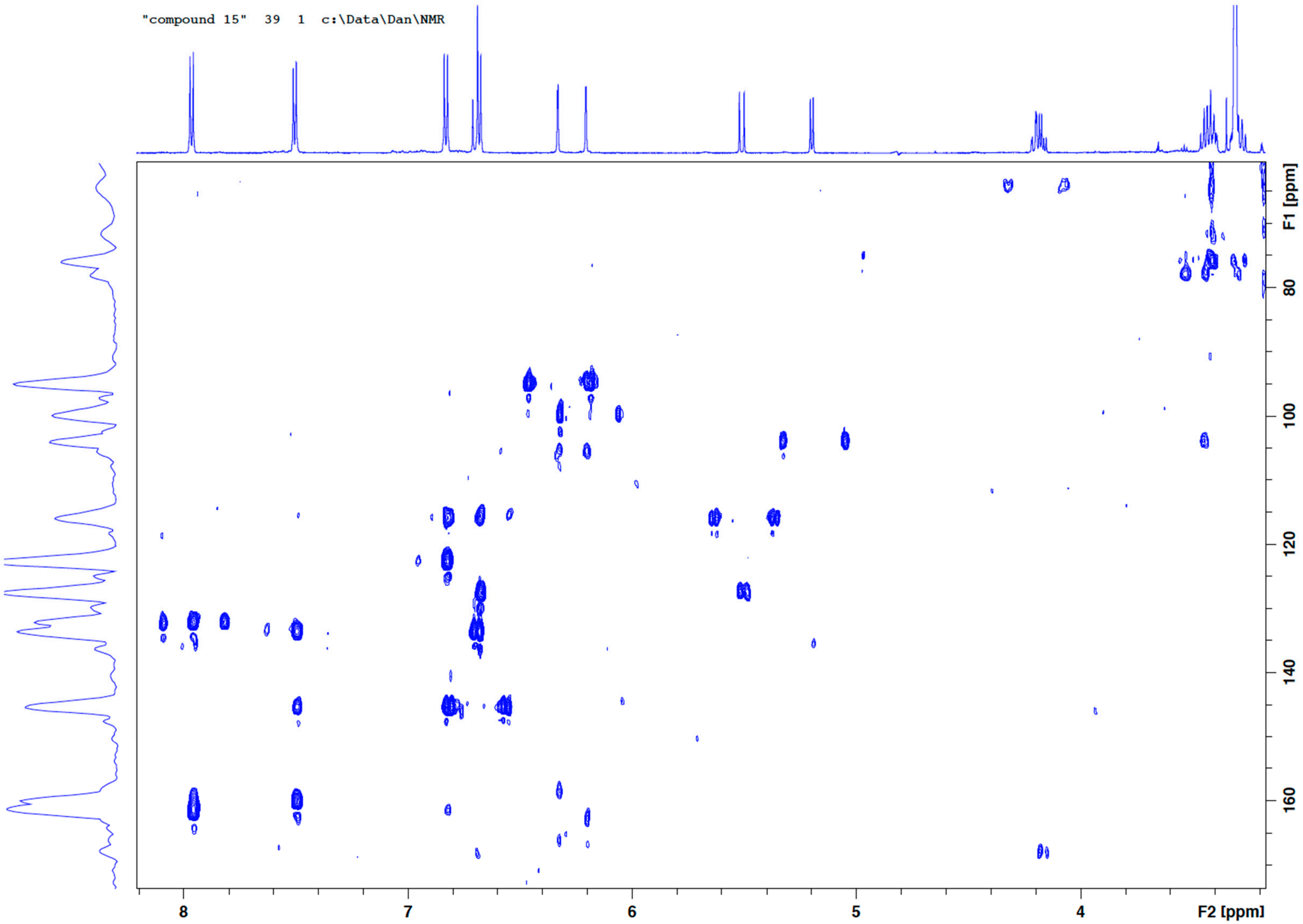

(**c**)

Figure S1. (**a**) ^{1}H NMR of compound **15** acquired in the HPLC-HRMS-SPE-NMR; (**b**) ^{1}H-^{13}C HSQC of compound **15** acquired in the HPLC-HRMS-SPE-NMR; (**c**) ^{1}H-^{13}C HMBC of compound **15** acquired in the HPLC-HRMS-SPE-NMR.

References for Table S1 (Numbering the Same as in the Published Paper)

[10]. Wubshet, S.G.; Schmidt, J.S.; Wiese, S.; Staerk, D. High-resolution screening combined with HPLC-HRMS-SPE-NMR for identification of potential health-promoting constituents in sea aster and searocket—New nordic food ingredients. *J. Agric. Food Chem.* **2013**, *61*, 8616–8623.

[29]. Lee, E.J.; Kim, J.S.; Kim, H.P.; Lee, J.H.; Kang, S.S. Phenolic constituents from the flower buds of *Lonicera japonica* and their 5-lipoxygenase inhibitory activities. *Food Chem*. **2010**, *120*, 134–139.

[30]. Fuchs, C.; Spiteller, G. Rapid and easy identification of isomers of coumaroyl- and caffeoyl-D-quinic acid by gas chromatography mass spectrometry. *J. Mass. Spectrom*. **1996**, *31*, 602–608.

[31]. Chen, J.; Mangelinckx, S.; Ma, L.; Wang, Z.; Li, W.; De Kimpe, N. Caffeoylquinic acid derivatives isolated from the aerial parts of *Gynura divaricata* and their yeast alpha-glucosidase and PTP1B inhibitory activity. *Fitoterapia* **2014**, *99*, 1–6.

[32]. Kong, C.S.; Kim, J.A.; Qian, Z.J.; Kim, Y.A.; Lee, J.I.; Kim, S.K.; Nam, T.J.; Seo, Y. Protective effect of isorhamnetin 3-O-β-D-glucopyranoside from *Salicornia herbacea* against oxidation-induced cell damage. *Food Chem. Toxicol*. **2009**, *47*, 1914–1920.

[33]. Calzada, F.; Cedillo-Rivera, R.; Mata, R. Antiprotozoal activity of the constituents of *Conyza filaginoides*. *J. Nat. Prod*. **2001**, *64*, 671–673.

[34]. Rashed, K.; Sahuc, M.E.; Deloison, G.; Calland, N.; Brodin, P.; Rouille, Y.; Seron, K. Potent antiviral activity of *Solanum rantonnetii* and the isolated compounds against hepatitis C virus *in vitro*. *J. Funct. Foods* **2014**, *11*, 185–191.

[35]. Soares, A.C.F.; Silva, A.N.; Matos, P.M.; da Silva, E.H.; Heleno, V.C.G.; Lopes, N.P.; Lopes, J.L.C.; Sass, D.C. Complete ^{1}H and ^{13}C NMR structural assignments for a group of four goyazensolide-type furanoheliangolides. *Quim. Nova* **2012**, *35*, 2205–2207.

[36]. Wei, X.Y.; Huang, H.J.; Wu, P.; Cao, H.L.; Ye, W.H. Phenolic constituents from *Mikania micrantha*. *Biochem. Syst. Ecol*. **2004**, *32*, 1091–1096.

[37]. Yi, B.; Hu, L.; Mei, W.; Zhou, K.; Wang, H.; Luo, Y.; Wei, X.; Dai, H. Antioxidant phenolic compounds of cassava (manihot esculenta) from Hainan. *Molecules* **2011**, *16*, 10157–10167.

[38]. Lee, D.Y.; Shrestha, S.; Seo, W.D.; Lee, M.H.; Jeong, T.S.; Cho, J.H.; Song, Y.C.; Kang, H.W.; Rho, Y.D.; Baek, N.I. Structural and quantitative analysis of antioxidant and low-density lipoprotein-antioxidant flavonoids from the grains of sugary rice. *J. Med. Food* **2012**, *15*, 399–405.

[39]. Wang, Y.; Hamburger, M.; Gueho, J.; Hostettmann, K. Cyclohexanecarboxylic-acid derivatives from *Psiadia trinervia*. *Helv. Chim. Acta* **1992**, *75*, 269–275.

[40]. Timmers, M.; Urban, S. On-line (HPLC-NMR) and off-line phytochemical profiling of the australian plant, *Lasiopetalum macrophyllum*. *Nat. Prod. Commun*. **2012**, *7*, 551–560.

[41]. Jou, S.J.; Chen, C.H.; Guh, J.H.; Lee, C.N.; Lee, S.S. Flavonol glycosides and cytotoxic triterpenoids from *Alphitonia philippinensis*. *J. Chin. Chem. Soc. Taip*. **2004**, *51*, 827-834.
